# Protocol of the interdisciplinary and intersectoral randomized controlled trial in Parkinson patients in a specialized network in Germany: the INSPIRE Trial

**DOI:** 10.1186/s42466-026-00476-6

**Published:** 2026-04-27

**Authors:** Susanne A. Schneider, Sandra Paryjas, Franziska Beyer, Jan-Philipp Bach, Simon Baudrexel, Bernhard Baier, Moaz Al Ajia, Ilona Csoti, Klaus Gehring, Gabriel Gonzalez Escamilla, Damian M. Herz, Markus Leisse, Leonore Köhler, Thea-Marie Krause, Daniel Martens, Uwe Meier, Maike Scheipers, Julia Schiffer, Nils Schröter, Rieke Schulz, Stefan Specht, Manfred Erbsland, Renate Stemmer, Sergiu Groppa

**Affiliations:** 1 Neuroimaging Center (NIC), INSPIRE-PNRM+, Universitätsmedizin der Johannes Gutenberg Mainz, Langenbeckstrasse 1, Gebäude: 308C, Mainz, 55131 Germany; 2 Klinik für Neurologie, Universitätsklinikum des Saarlandes, Homburg, Germany; 3 Klinik für Neurologie, Praxis für Neurologie & Psychiatrie Gernsheim, Philipps-Universität Marburg sowie, Marburg, Germany; 4 Klinik für Neurologie, Universitätsklinikum Frankfurt, Schleusenweg 2-16, 60528 Germany; 5 Edith-Stein-Fachklinik, Wiesenstr. 25, Bad Bergzabern, 76887 Germany; 6 NeuroImaging Center, University Medical Centre of the Johannes Gutenberg University Mainz, Mainz, Germany; 7 5Getrudis-Klinik Biskirchen, Parkinson Fachklinik, Leun-Biskirchen, Germany; 8 Neurozentrum am Klosterforst GmbH, Itzehoe, Germany; 9 Abteilung für Neurologie, Universitätsklinik Heidelberg, Heidelberg, Germany; 10 Department of Neurology, MEDIAN Reha-Zentrum Bernkastel-Kues Klinik Burg Landshut, Bernkastel-Kues, Kueser Plateau Germany; 11 Hochschule für Wirtschaft und Gesellschaft Ludwigshafen, Institut für Management, Ökonomie und Versorgung, im Gesundheitsbereich (IMÖVG), Ludwigshafen am Rhein, Germany; 12 Pathways Public Health GmbH, Neustädtische Kirchstraße 8, Berlin, 10117 Germany; 13 Neuro-Centrum Grevenbroich, Grevenbroich, Germany; 14 Klinik für Neurologie, Universitätsmedizin der Johannes Gutenberg Mainz, Langenbeckstrasse 1, Mainz, 55131 Germany; 15 Neurologicum Griesheim, Griesheim, Germany; 16 Fachbereich Gesundheit und Pflege, Katholische Hochschule Mainz, Saarstr. 3, Mainz, Germany

## Abstract

**Abstract:**

INSPIRE-PNRM+ (short for InterdiSciPlinary and InteRsectoral telemedIcal Evaluation, Coordination and Therapy at the ParkinsonNet RheinMain+) is an one-year, multicentre, interventional, open-label, two-arm, 1:1 randomized controlled trial (RCT) with the goal to improve the quality of care for Parkinson’s disease (PD) patients outside of a specialized clinical context. The study is funded by the German Innovation Fund mandated by the Federal Joint Committee (G-BA). The three core characteristics are (1) the employment of specially trained advanced practice nurses (APN) in an (2) interdisciplinary network and (3) the use of a specifically designed digital platform. This platform serves for documentation and communication, thereby helping patients receive individualized, guideline-based treatment and support in adapting their lives to the effects of the disease. 844 patients will be recruited. They will be divided into two groups: a “usual care” group which continues traditional care (i.e., usually consultation with a neurologist in private practice) vs. the “intervention” group which will have APN contact every three months or more frequently as needed over the period of 12 months. Patients’ quality of life serves as primary outcome measure, combined with a process evaluation and health economic analysis (cost-utility analysis (CUA)). Recruitment was ongoing at time of submission of this paper. This innovative study is the first in Germany where APNs are dedicated to PD care. The study may serve as blueprint when incorporating APNs into routine healthcare, similar to other countries.

**Trial registration:**

The study has been registered at www.clinicaltrials.gov, NCT06479083. Registered June 27, 2024. https://clinicaltrials.gov/study/NCT06479083?term=INSPIRE%20Parkinson&rank=1.

## Introduction

With a prevalence of around 0.35%, accounting for about 300.000 people in Germany [1], Parkinson’s disease (PD) is the second most common neurodegenerative disease after Alzheimer’s dementia, often leading to disability, the need for care, reduced quality of life and premature death.

Cardinal clinical symptoms include motor impairment with lack of movement (akinesia), muscle stiffness (rigidity), tremor and impaired postural stability with gait impairment with a risk of falls [2]. These typically start gradually and unilaterally; however, they soon evolve to bilateral involvement affecting day to day life and independence. Additional symptoms may include difficulties in speaking and swallowing, disturbances in autonomic function (e.g. blood pressure and digestion), sleep disorders, depression and mental impairment including dementia.

In addition to idiopathic Parkinson’s disease (PD), atypical parkinsonian syndromes are part of the broader spectrum of parkinsonian disorders. These include dementia with Lewy bodies (DLB), multiple system atrophy (MSA), progressive supranuclear palsy (PSP), and corticobasal degeneration (CBD). Compared to idiopathic PD, atypical parkinsonian syndromes generally exhibit a more rapid progression, involve additional brain regions, and show a limited or poor response to levodopa therapy [3].

Treatment of PD and atypical parkinsonian disorders remains symptomatic. There are no causative or neuroprotective therapies available, so patients are faced to combine multiple drugs from different drug classes, non-pharmacological interventions, and, in advanced stages, pump therapies or deep brain stimulation in some cases [4]. Especially as the disease progresses treatment becomes increasingly complex requiring multidisciplinary, personalized care that considers each patient’s medical, nursing and social situation [5]. Furthermore, with the forecast of change in the demographic age structure and increasing life expectancy, the expected number of people affected by a Parkinsonian disorder and the associated costs to the healthcare system are expected to rise further in the future [6, 7]. This poses an urgent unmet medical need.

However, integrated PD care networks are scarce making the access to coordinated multidisciplinary teams which provide specialized PD care a limited option. Instead, many patients experience lack of guidance in a complicated healthcare system [8].

Some countries such as the US, Australia, Canada and the Netherlands have incorporated nurse specialists/advanced practice nurses (e.g. PD nurses who are qualified at Master’s level) as part of their healthcare landscape [9]. In Germany, multidisciplinary teams dedicated to oncological disease have been successful in the last decade. However, there are less than a dozen specialised care networks for other disorders. To our knowledge there are none in Germany dedicated to PD which employ APNs.

In the attempt to improve healthcare provided under the statutory health insurance system and to change the landscape towards an optimized, effective, efficient and needs-based care for people with PD and atypical Parkinsonian syndromes we carefully planned the INSPIRE-PNRM+ study, funded by the German Innovation Fund mandated by the Federal Joint Committee (G-BA). The protocol is published herein.

The innovative protocol was developed through a close collaboration between the Sponsor, Key Opinion Leaders, representatives of the Patient Community, and National Regulatory Authorities. Methodological and statistical concerns were discussed, with an emphasis on selecting a primary outcome measure that focuses on the aspects of the diseases that are relevant and meaningful to patients and affect patient’s quality of life. The pathway to the study approval was facilitated by frequent communication between all parties, collaborative adaptation of study methodology and statistical approaches ensuring that feasibility and meaningfulness.

### Study aim of INSPIRE-PNRM + and study design

As the acronym suggests, aim of INSPIRE-PNRM + is the InterdiSciPlinary and InteRsectoral telemedIcal Evaluation, Coordination and Therapy at the ParkinsonNet RheinMain + in order to overcome insufficient integration of concomitant therapies into personalized treatment plans and interlinking treatment chains. This is an interventional, open-label, randomized controlled trial (RCT) with two arms, designed to assess efficacy over a one-year period, combined with a process evaluation and health economic analysis (cost-utility analysis (CUA)).

INSPIRE-PNRM + has three key core components:

(1) *Advanced Practice Nurses*.

The core component is the employment of advanced practice nurses (APNs), i.e. master level qualified-nurses with at least 2 years of professional experience in general nursing as well as a specialization on PD. For the purpose of this project, APNs received structured, focussed training (300 training hours) about clinical aspects, telehealth/telemedical care, general nursing, and APN-role development. This yielded in the qualification to initiate and conduct treatments according to current standards and guidelines and in the case of measures subject to prescription carry these out under telemedical supervision. Notably, across Germany there are yet no standardized training requirements for PD-dedicated non-physician healthcare providers. Since 2006, part-time training for Parkinson Nurses exists, which usually consists of four two-day modules and two weeks of clinical observation spread over one year. However, compared to this, APNs, as they are enrolled in the INSPIRE-PNRM+ programme, have much higher qualifications as described above.

(2) *Implementation of an optimized interdisciplinary and transsectoral network*.

The second central component is to integrate INSPIRE-PNRM+ into and thereby improve existing interdisciplinary and transsectoral outpatient care structures. INSPIRE-PNRM+ connects numerous care providers, i.e. 3 university hospitals (acute care), 1 rehabilitation/post-acute care hospital, 33 neurological specialists/consultant neurologists in private practice and, of course, 10 APNs. In a broader scope, there are a total of 56 partners with the local Parkinson Network which include allied health professionals (e.g. physiotherapists, occupational therapists, speech therapists), pharmacies, additional hospitals as well as PD patient organisations.

(3) *Implementation of a telehealth platform*.

The third central component of the project is the use of the telemedicine platform provided by the Zentrum für Telemedizin (ZTM) specifically for this purpose which contains an electronic case file and fosters communication and meetings between patients (e.g. for video calls) as well as between all healthcare providers (e.g. for authorization of proposed management decisions).

### Study oversight and ethics

The study is conducted in accordance with the International Conference for Harmonisation - Good Clinical Practice Guideline, the General Data Protection Regulator, and the Declaration of Helsinki. The study has been approved by the ethics committees of each participating center, primarily the University of Mainz (ref. no. 2024–17454). All participants gave written informed consent.

#### Funding

INSPIRE-PNRM + is funded by the Innovation Fund (ref. no. 01NVF22107) according to the legislation’s intended purpose to ensure the development of easily accessible high-quality medical care and integrated care programs. The funding body had no influence on design of the study and collection, analysis, and interpretation of data and in writing the manuscript.

#### Study population and eligibility criteria

The study population consists of people affected by PD or an atypical parkinsonian syndrome. Inclusion and exclusion criteria are shown in Table 1. In accordance with the funding agreement, only individuals covered by statutory (public) health insurance were eligible to participate, while those with private insurance were excluded. Two statutory health insurance providers will supply data on the economic impact of the intervention through a Selektivvertrag (selective contract) agreement.

The study will enrol approximately 844 patients (see below). First patient in was in July 1, 2024.

### Study centers/collaboration partners

The PNRM+ network fosters collaboration between numerous partners.

### Recruitment and patient involvement

Patients are recruited via personal correspondence, routine care appointments, and referrals. In addition, there is tremendous collaboration and support from patient organizations representing the Parkinson communities. 

The principal investigator at each study site is responsible for obtaining informed consent for each patient. All eligible patients who agree to participate in the study are provided with a full verbal explanation of the trial and the patient information sheet. This includes detailed information about the rationale, design, and personal implications of the study.

### Study intervention procedures

All patients undergo an initial comprehensive assessment by the APN of their health and care situation in their homes (baseline visit; see Figure 1). Assessments includes a motor exam (UPDRS), structured interviews regarding non-motor symptoms, the familial and professional social and psychosocial situation, the living and housing situation (including support structures, accessibility), and previous and current treatment and therapies, and standardized questionnaires including the International Consortium for Health Outcome Measurements (ICHOM) as part of the internationally established battery for recording the patient-centered evaluation, quality of life questionnaire (PDQ39). All assessments are carried out independently by the APN. Patients are then 1:1 randomized into either the intervention group or the control group. The control group does not receive further APN input until the final visit at 12 months. For patients from the intervention group the APN draws up a person-centered and evidence-based treatment plan. They ensure care is provided in line with requirements and guidelines or initiates appropriate measures in accordance with the treating neurologists within the specialized PNRM+. If the APN recommends a prescription for a subject, these are passed on to the treating neurologist (nVF4) in accordance with current legislation. The APN is responsible for monitoring the treatment plan, evaluates it quarterly in the follow-up (nVF6) and implements adjustments as necessary. The APN also recommends which level of care (private practice, board, hospital outpatient care) is deemed necessary to provide patients with the care they need without delay. 

Through the PNRM+ network the APN plans and coordinates the cross-sectoral initiation of necessary treatment steps and provides those involved with patient-relevant information (nVF8). APNs also have the opportunity to easily consult with a movement disorders expert from one of the University Medical Centers on an individual patient basis (nVF12) or initiate a geriatric/psychiatric board (nVF9; nVF10) if they notice a deterioration in the patient's state of health. Here, too, the APN can provide first aid on a situational basis. 

Telehealth-supported communication with patients plays a major role in the implementation of treatment plans. Thus, as part of disease education (nVF5), patients and their care givers receive comprehensive, needs-based, participatory advice and training on various aspects related to the disease, treatment or life situation including coping strategies tailored to individual needs from APNs. This allows patients sufficient time to ask questions, enabled “shared decision making” that consolidates health literacy and strengthened self-efficacy of patients. In addition to these care services, on-demand services are established with the intent that urgent health requests can be addressed by the APN making it obsolete to call the national emergency hotline (i.e, 112 equivalent to 911). 

At 12 months, both the intervention and the control group undergo a final visit to reevaluate the patient’s situation and disease parameters. These and other clinical measures will be analysed together with numerous health economic parameters (see Table 2) in order to answer the main question: did the additional innovative fusion of three components result in added value for patients?

### Statistics: case number calculation

The PDQ-39, used as a measure of quality of life, is based on a Likert scale and can therefore be treated as providing metric-level data. To estimate the required sample size based on the research hypothesis, a two-sample t-test for independent groups was used to compare means. Cohen’s d served as the a priori effect size, reflecting the standardized mean difference. An approximated value of d = (5 − 2)/14.48 = 0.207 was used. The significance level was set at α = 0.05. According to Horváth et al. (2017), the minimally clinically important difference (MCID) for the PDQ-39 is −4.72 points (12). Based on this MCID, the assumed effect size, a statistical power of 80%, and an estimated dropout rate of 20%, the required total number of participants to complete the intervention was calculated to be 620. The sample size calculation was performed using G*Power (version 3.1).

### Study endpoints

Quality of life reflected by the PDQ-39 will serve as primary endpoint. Secondary are listed in Table 3.

### Trial status

At the time of manuscript submission, the protocol for the INSPIRE-PNRM+ has been accepted/approved (Date, March 7, 2024); recruitment is ongoing. More than 500 patients have been enrolled and are actively treated according to protocol. 

## Discussion

INSPIRE-PNRM+ is an innovative cross-sector study aimed at improving the quality of care for Parkinson's patients outside of a specialized clinical context and at ensuring an efficient allocation of resources and a professional exchange between all interprofessional healthcare professionals.

INSPIRE-PNRM+ has three unique components: From the clinical point of view, the study assesses the impact of an APN intervention versus usual care on PD management. To elaborate, the care is provided by specially trained APN enabling patients to receive individualized guideline-based treatments and support in adapting their life situation to the consequences of the disease. 

APNs have many broad-based skills, at the interface of nursing and medical practice. As critical member of multidisciplinary teams their know-how allows APNs to monitor symptoms, interpret exams, provide advice and psycho-social support, recommend additional investigations and adjustment of therapy including prescribed medication, coordinate care and make complex decisions - in the case of measures requiring a prescription approved by a neurologist. APNs are an integral part of the healthcare system in the UK, US and Australia, where institutional protocols related to advanced nursing practice have been formulated. However, systematic data on the impact of specialist nurse care in the context of PD is limited including few randomised controlled trials from the UK [10−12], the US13, Germany [14] and the Netherlands [15, 16] for review see [9]. Key findings were increased adherence to PD quality of care indicators, improved sense of wellbeing and high satisfaction of patients and healthcare providers. 

The second characteristic of the INSPIRE-PNRM+ study is the digital platform for documentation and communication, specifically designed for this purpose. The platform enables remote monitoring, seamless case-specific information exchange among healthcare providers, timely decision-making and ultimately improved patient outcomes. Thus, the digital solution serves an important cornerstone for the effective, continuous and personalized PD management.

One weakness of the study is the relative short intervention period of 12 months. A longer period would likely better reflect the impact of APN intervention in PD management and treatment. Second, the open-label character of this study may influence the reactions and behaviors of patients and healthcare providers leading to performance bias. Third, participants in the current study had early or mid-stage PD. Patients with concomitant dementia, defined by MOCA<19, did not meet inclusion criteria. Future studies focussing on these patients are needed, given that treatment then becomes even more complex and challenging for care-givers. 

In summary, the concept of the INSPIRE-PNRM+ study is novel for Germany, building on longstanding international experience where APN are integral part of PD care. Positive evaluation of this a new model of healthcare delivery, will provide the basis for long-term integration and implementation into standard care with APN as part of a multidisciplinary Clinical Care Team [17]. In parallel, standardized training opportunities tailored to the care priorities of PD-patients will be needed [13]. 

**Table 1:** Inclusion and exclusion criteria

Inclusion criteria	Exclusion criteria
Age: 30–85 years	Profound dementia (MOCA < 19)
PD (ICD: G20.-) or atypical PD (ICD: G23.-), according to the current diagnostic criteria of the Movement Disorder Society	Severe depression
Resident of Saarland, Hesse and Rhineland-Palatinate (INSPIRE-PNRM+ region)	Psychoses or other psychiatric or medical comorbidities that could interfere with study participation (e.g. tumour diseases with limited life expectancy, dialysis requirement, etc.)
Ability to consent	Drug or alcohol addiction
Insurance cover with a public health insurance company	Pregnancy or breast-feeding
	Simultaneous participation in another interventional treatment study
	Illiteracy or insufficient language skills to complete the questionnaires
	Insurance cover with a private health insurance company

**Table 2:** Key research questions

Research questions	Related outcome measures
Differences in the change in Parkinson’s-specific quality of life after 12 months	PDQ-39 sum index (primary endpoint)
Differences in the change in motor symptoms after 12 months	MDS-UPDRS, part 3
Differences in the change in disease-overarching quality of life after 12 months	EQ-5D-5 L
Differences in the expression of clinical, cognitive and social variables	Self-Administered Comorbidity Questionnaire (SCQ); Hoehn & Yahr (H&Y); Life-Space Assessment Scale (LSA); Montreal Cognitive Assessment (MOCA); The Parkinson Anxiety Scale (PAS); Beck Depression Inventory (BDI); The Starkstein Apathy Scale (SAS); − Scales for Outcomes in Parkinson’s disease (SCOPA); Zarit Burden Interview (ZBI); Involvement Evaluation Questionnaire (IEQ); Tasmanian Test (TAS Test): assessment of cognitive and motor function; Health Education Impact Questionnaire (heiQ V. 3) (explorative analysis)
Differences in statutory health insurance resource use over 12 months (health economic analysis)	Δcost: Δbenefit value between the intervention group and the control group over the 12-month observation period, considering cost components such as allocation of medications, allocation of remedies and aids

## Data Availability

All data and material are present within the main manuscript.

## Abbreviations

APN: Advanced practice nurses
BDI: Beck Depression Inventory
CBD: Corticobasal degeneration
CUA: Cost-utility analysis
DLB: Dementia with Lewy bodies
EQ-5D-5L: EuroQol 5-Dimension 5-Level questionnaire
G-BA: German Innovation Fund
H&Y: Hoehn & Yahr
heiQ V. 3: Health Education Impact Questionnaire
ICHOM: International Consortium for Health Outcome Measurements
IEQ: Involvement Evaluation Questionnaire
INSPIRE-PNRM+: InterdiSciPlinary and InteRsectoral telemedIcal Evaluation, Coordination and Therapy at the ParkinsonNet RheinMain+
LSA: Life-Space Assessment Scale
MDS-UPDRS: Movement Disorder Society–Unified Parkinson’s Disease Rating Scale
PAS: The Parkinson Anxiety Scale
PSP: Progressive supranuclear palsy
MCID: Minimally clinically important difference
MOCA: Montreal Cognitive Assessment
MSA: Multiple system atrophy
PD: Parkinson’s disease
PDQ39: Parkinson’s Disease quality of life Questionnaire 39
RCT: Randomized controlled trial
SAS: Starkstein Apathy Scale
SCOPA: Scales for Outcomes in Parkinson’s disease
Self: Administered Comorbidity Questionnaire
TAS: Tasmanian Test
ZBI: Zarit Burden Interview
ZTM: Zentrum für Telemedizin
